# Surveillance of *Aedes aegypti*: Comparison of House Index with Four Alternative Traps

**DOI:** 10.1371/journal.pntd.0003475

**Published:** 2015-02-10

**Authors:** Claudia T. Codeço, Arthur W. S. Lima, Simone C. Araújo, José Bento P. Lima, Rafael Maciel-de-Freitas, Nildimar A. Honório, Allan K. R. Galardo, Ima A. Braga, Giovanini E. Coelho, Denise Valle

**Affiliations:** 1 Programa de Computação Científica, Fundação Oswaldo Cruz, Rio de Janeiro, Brazil; 2 Laboratório de Fisiologia e Controle de Artrópodes Vetores, Fundação Oswaldo Cruz, Rio de Janeiro, Brazil; 3 Laboratório de Transmissores de Hematozoários, Fundação Oswaldo Cruz, Rio de Janeiro, Brazil; 4 Laboratório de Entomologia Médica, Instituto de Pesquisas Científicas e Tecnológicas do Estado do Amapá-IEPA, Amapá, Brazil; 5 Secretaria de Vigilância em Saúde, Ministério da Saúde, Brasilia, Brazil; 6 Laboratório de Biologia Molecular de Flavivírus, Fundação Oswaldo Cruz, Rio de Janeiro, and Instituto Nacional de Ciência e Tecnologia—Entomologia Molecular, Brazil; North Carolina State University, UNITED STATES

## Abstract

**Introduction:**

The mosquito *Aedes aegypti*, vector of dengue, chikungunya and yellow fever viruses, is an important target of vector control programs in tropical countries. Most mosquito surveillance programs are still based on the traditional household larval surveys, despite the availability of new trapping devices. We report the results of a multicentric entomological survey using four types of traps, besides the larval survey, to compare the entomological indices generated by these different surveillance tools in terms of their sensitivity to detect mosquito density variation.

**Methods:**

The study was conducted in five mid-sized cities, representing variations of tropical climate regimens. Surveillance schemes using traps for adults (BG-Sentinel, Adultrap and MosquiTRAP) or eggs (ovitraps) were applied monthly to three 1 km^2^ areas per city. Simultaneously, larval surveys were performed. Trap positivity and density indices in each area were calculated and regressed against meteorological variables to characterize the seasonal pattern of mosquito infestation in all cities, as measured by each of the four traps.

**Results:**

The House Index was consistently low in most cities, with median always 0. Traps rarely produced null indices, pointing to their greater sensitivity in detecting the presence of *Ae. aegypti* in comparison to the larval survey. Trap positivity indices tend to plateau at high mosquito densities. Despite this, both indices, positivity and density, agreed on the seasonality of mosquito abundance in all cities. Mosquito seasonality associated preferentially with temperature than with precipitation even in areas where temperature variation is small.

**Conclusions:**

All investigated traps performed better than the House Index in measuring the seasonal variation in mosquito abundance and should be considered as complements or alternatives to larval surveys. Choice between traps should further consider differences of cost and ease-of-use.

## Author Summary

Dengue vector surveillance programs regularly use household surveys for searching breeding sites that are positive for *Ae. aegypti* larvae. Infestation indices are calculated as the percentage of positive houses, or percentage of positive containers, and are used to guide control actions and to issue alerts. However, these indices are costly and prone to error due to variation in searching effort and the sometimes cryptic nature of the mosquito egg laying behavior. In the recent years, many devices for trapping mosquitoes were developed. One problem when deciding which trap to choose for surveillance is the lack of a gold standard to compare trap-based indices with. In this scenario, choice relies on the behavior of the trap indices, that is, how well they inform about mosquito population growth, decline, or spread. Our study compared infestation indices produced by four different trap schemes as well the immature survey. We observed that any trap is more sensitive in detecting the mosquito presence than the immature survey, and they are better in capturing the temporal variation in mosquito abundance as well. However, traps are very different in terms of cost, ease-of-use and sensitivity and trap choice should further consider these factors.

## Introduction

The mosquito *Aedes aegypti*, vector of dengue, chikungunya and yellow fever viruses, is an important target of vector control programs in tropical countries. Traditional *Ae. aegypti* surveillance is based on periodic household inspections for the presence of larvae-bearing containers, which inform health agents on the most productive breeding sites and trigger control strategies in the form of container removal or chemical treatment. Household surveys also provide measures of infestation in the form of House (HI) and Breteau indices (BI). Based on the former, risk of disease transmission is empirically defined as low if HI<1.0%, or high, if HI> = 4.0%, and these thresholds guide control initiatives [1].

House indices face many criticisms: household surveys are costly to be performed with the frequency required for surveillance; indices are highly dependent on both agent’s effort and householder availability; these surveys only provide qualitative measures of abundance as the number of immatures per container or premise is not computed, only their presence/absence; moreover, larval density is not a precise measurement of mosquito adult density, the stage involved in dengue virus transmission [2].

Traps are promising alternatives to larval surveys: they transfer the searching effort from the health agents to the mosquitoes themselves (this time saved allows more frequent surveys); and traps provide qualitative (% of positive traps) and quantitative (number of captures per trap) indices [2, 3].

There are currently several traps for *Ae. aegypti*, varying in their attractiveness, specificity to different mosquito life stages, ease-of-use, and cost. The classical trap is the patent-free ovitrap, a tool designed to attract and collect eggs of female mosquitoes searching places to oviposit. This trap, in use since 1965, is employed together with routine larval surveys in many countries, due to its high sensitivity and low cost [4–6]. When mosquito infestation is low, ovitraps are more sensitive for detecting *Ae. aegypti* presence than larval surveys [7]. Ovitraps have disadvantages as well: variation in the local availability of breeding sites may interfere with their attractiveness by affecting the probability of egg deposition in these traps, potentially impairing comparison among areas; the skipping oviposition behavior of *Aedes* females may also affect the number of eggs deposited in individual traps and the reliability of adult abundance estimation derived from egg counts.

Traps against adults are often presented as alternatives to the ovitrap and to larval surveys (see [2] for a review). In principle, the advantage of adult catching traps is to obtain estimates of the population that is directly involved in transmission. Currently available adult capturing traps are designed to attract only a subset of the adult population: the ovipositing females are attracted to Adultrap [8] and to sticky traps, like MosquiTRAP; host seeking females are attracted to the BioGent-Sentinel [9]. The reliability of the infestation indices will depend on a series of parameters, as efficiency, sensibility and specificity.

Attractiveness is an important issue as any trap has to compete for the“attention” of the target individuals [10]. Ovipositing traps compete with other breeding sites, and traps emulating a host compete with hosts themselves. The quality of a trap depends on how their attractiveness remains unchanged as the environment changes.

Traps against adults (and ovitraps) do not provide absolute measurements of the adult population (which should be expressed in“mosquitoes/area” or“mosquitoes/person” units) [11, 12]. Trap indices are relative measures of abundance, having“mosquitoes per trap” unit. Conversion from relative to absolute measurements requires assumptions regarding the area effectively covered by a trap. The development of statistical methods for estimating absolute mosquito abundance (mosquitoes/person or mosquitoes/premise) from adult trap data is a recent subject [13].

One problem when deciding which trap to choose for surveillance is the lack of a gold standard to compare trap-based indices with. In this scenario, choice relies on the behavior of the trap indices, that is, how well they inform about mosquito population growth, decline, or spread. Ideally, traps’ statistical properties (sensitivity, specificity) should be invariant (that is, not influenced by anything but fluctuations in mosquito abundance), and consistently behave under both low and high abundances.

Here, we present the results of a two-year study designed in the context of a routine *Ae. aegypti* surveillance program to compare the standard larval survey and four traps: ovitrap, MosquiTRAP, Adultrap and BioGent-Sentinel. Five cities representing four climatically distinct dengue endemic regions in Brazil were simultaneously monitored by the different traps. We investigated the temporal consistence of the entomological indices produced by the different traps, comparing positivity and density indices, and trap indices versus larval indices.

## Methods

### Study areas

**Figure 1:**
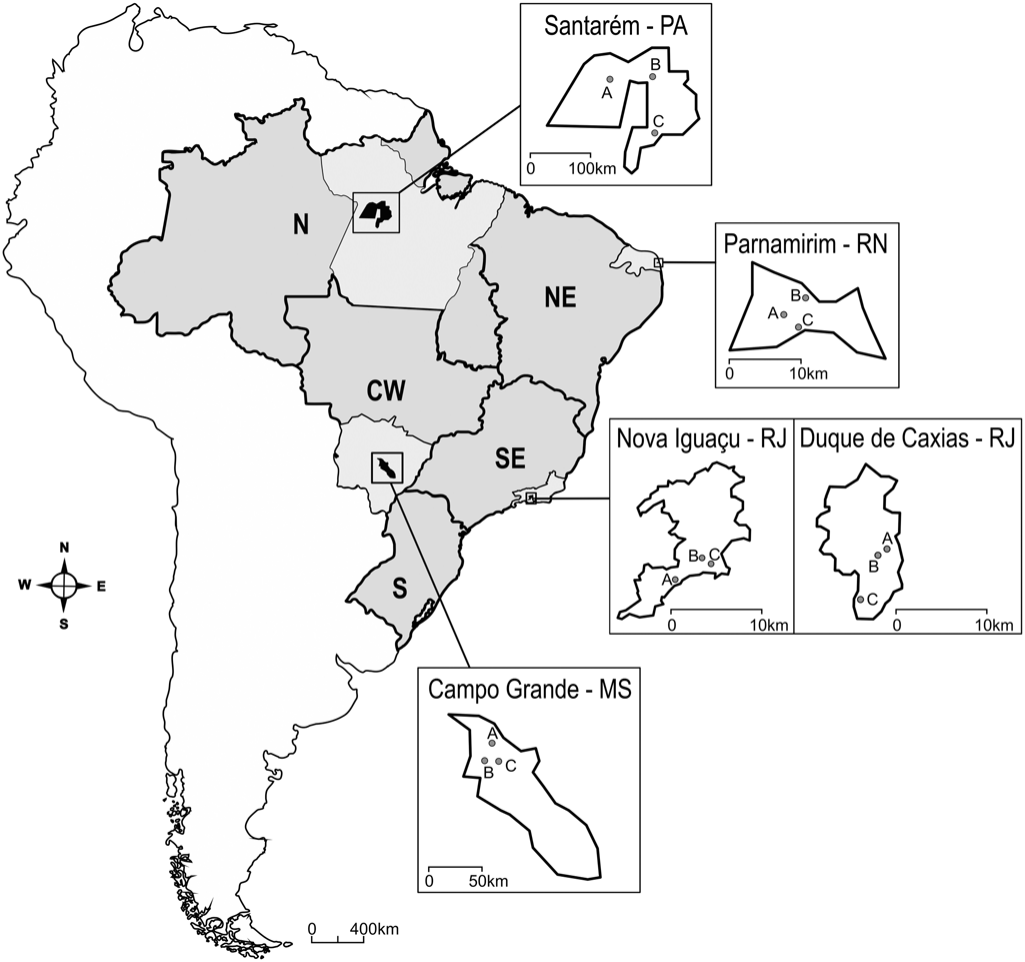
Map of Brazil with the location of the five cities included in the study. For each city in the map, A, B and C refer to the 1 km^2^ areas where traps were installed. Letters beside city names account for the respective States: Santarem—Pará (PA), Parnamirim—Rio Grande do Norte (RN), Nova Iguaçu and Duque de Caxias—Rio de Janeiro (RJ) and Campo Grande—Mato Grosso do Sul (MS). References to the different Brazilian Regions are placed directly on the map: N—North, NE—Northeast, SE—Southeast, S—South, CW—Central-West.

**Figure 2:**
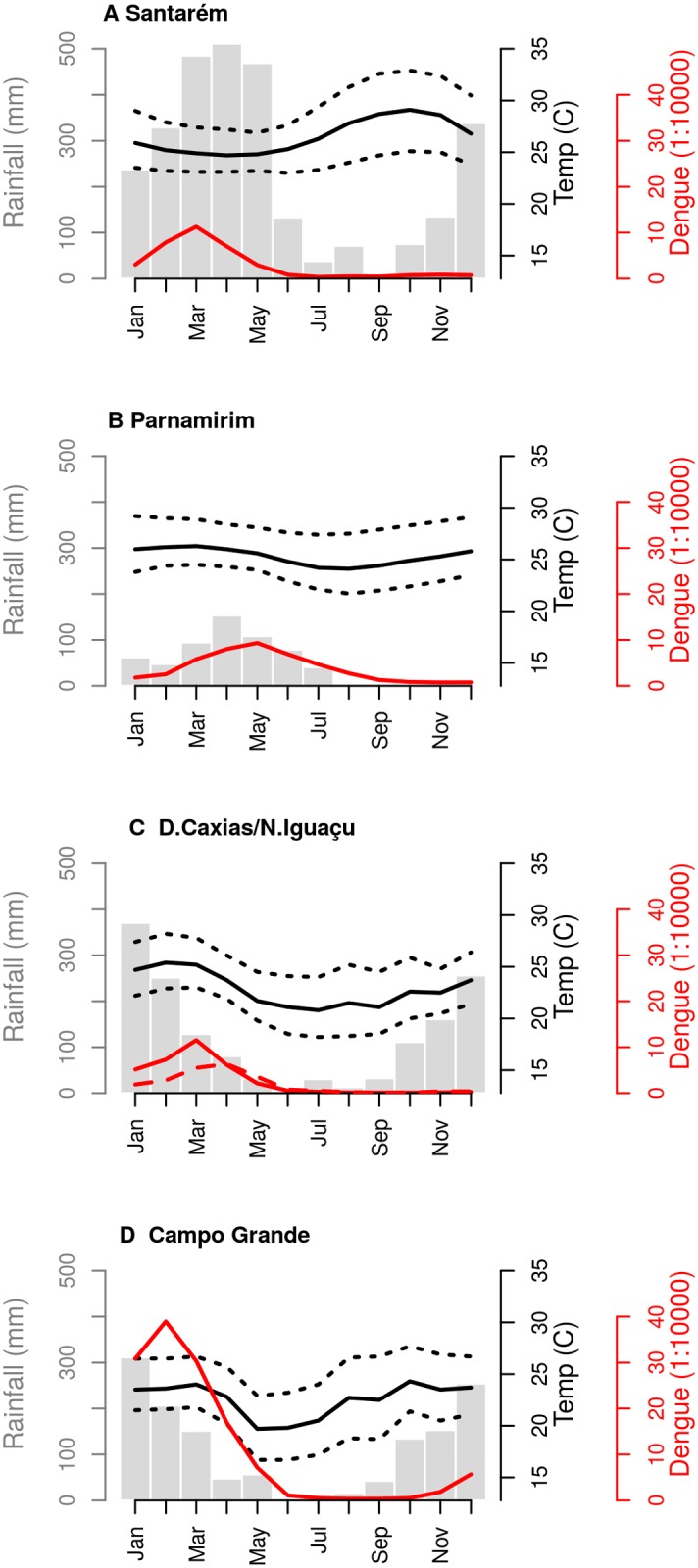
Climate and dengue seasonality in each study site. Campo Grande (CGR), Parnamirim (PNM), Santarém (STR) and the neighbor cities of Duque de Caxias and Nova Iguaçu (DQC-NIG). Grey bars: monthly accumulated rainfall; solid and dotted black lines: mean, minimum and maximum monthly temperatures; red lines: 2001–2009 average monthly dengue incidence (cases per 10,000 inhabitants). Dashed red line: dengue incidence in NIG. Data sources: Dengue incidence http://dtr2004.saude.gov.br/sinanweb/), population (http://cidades.ibge.gov.br/xtras/home.php), meteorological data (SISAM http://sisam.cptec.inpe.br/sisam/).

Brazil extends from the Equator to sub-tropical latitudes (05°15′05″N to 33°45′09″S). Except for the southernmost region, dengue is endemic throughout the country. Within this dengue endemic zone, five mid sized cities representing different climate regimes were chosen for this study (Figs. 1 and 2). In each city, three non-adjacent 1 km^2^ areas with roughly equivalent demography and geographical characteristics were chosen as study sites, totalizing 15 study areas:

**Santarém (STR), North Region**. 2°26′35″S, 54°42′29″W, 20m elevation. Located in the Amazon region, this city has a tropical climate with no dry season(Köppen-Geiger type Af), wit warm temperatures year around (23–33°C) and a monsoon season from January to May when precipitation may reach 500 mm in a single month. The dengue fever season tends to coincide with the monsoon period.

**Parnamirim (PNM), Northeast Region**. 5°54′56″ S, 35°15′46″ W, 26m elevation. It is a tropical city (Köppen-Geiger type As), with warm climate year around (summer: 24–29°C, winter: 22–27°C). Compared to Santarém, Parnamirim is considerably drier, with monthly precipitation reaching its maximum in April-May, with less than 200 mm / month. Dengue transmission intensifies during the wet-warmer season.

**Duque de Caxias (DQC) and Nova Iguaçu (NIG), Southeast Region**. 22°47′08″ S, 43°18′42″, 7m elevation. Adjacent cities located in a lowland region, with tropical climate (Köppen-Geiger type Aw/Am). Heavy precipitation occurs during summer (December to February), with occasional floods. Temperature differences between summer and winter are more pronounced than in the North and Northeast Regions. Dengue incidence often peaks in March, at the end of the rainy season. Due to logistic reasons, survey was conducted in DQC in the first year and shifted to NIG during the second year. Both cities belong to the same metropolitan area, are similar in demography, land use and geographical features (S1 Table in S1 Text).

**Campo Grande (CGR), Central-West Region**. 20°26′34″ S, 54°38′47″ W, 600m elevation. With a highland tropical climate (Köppen-Geiger type Aw), this city presents the highest temperature and precipitation amplitudes among the localities under study. The winters are particularly dry and cool (Fig. 2). Dengue incidence historically peaks at late summer, at the end of the rainy season.

### *Ae. aegypti* surveillance

Four traps were employed: Adultrap (ADT), BG-Sentinel (BGS), MosquiTRAP (MQT) and Ovitrap (OVT). Simultaneously, larval surveys were performed according to the routine policies of the Brazilian Dengue Control Program [1]. In all cases, trap installation or larval survey depended on householders’ oral consent. To ensure good spatial coverage, each 1 km^2^ area was subdivided into 4 sub-areas of 250×250 m and 1/4 of the traps was installed in each sub-area. To select houses, a random set of geographical coordinates was taken and once in the field, the closest house was chosen. The number of traps installed per km^2^ varied between trap types and seek to obey their manufacturer’s recommendations or, in its absence, previous recommendations from the scientific literature [14].

Except for DQC and NIG, in each locality, fieldwork lasted 24 months (details in S1 Table in S1 Text) with monthly samplings. In DQC and NIG, the survey lasted 12 months each. A brief description of the main characteristics of each trap, as well as the amount and schedule of traps installation in each 1 km^2^ area are detailed below.

**Adultrap (ADT)**. Adultrap is designed to capture gravid *Ae. aegypti* females during oviposition, using water as its principal attractant. A large hole on the top is the main entrance for attracted females that become trapped in the interior chamber. Water remains confined in a compartment at the bottom of the trap that cannot be reached by trapped mosquitoes, avoiding egg laying [15].

Adultrap was tested using two different approaches: exposure of 240 trap units /km^2^ during 24 hours (in the first year) or 100 units/km^2^ during 4 days (in the second year). Although the first approach is the one recommended by the manufacturer, the percentage of positive traps never reached 5% in the first year. Modifications of the protocol aimed at improving the surveillance ability of this trap.

**BG-Sentinel (BGS)**. It is a collapsible bucket with a white gauze covering its opening. In the middle of the gauze cover, there is a black tube through which a down flow is created by an electric power exhauster fan that captures mosquitoes flying in the vicinity of the opening into a catch bag. An attractant (BG-Lure) releases sinthetic human skin odors that attracts preferentially host-seeking females [10].

A total of 24 BG-Sentinels were installed during 24 hours per 1 km^2^ area. Differently from the other traps, BGS were installed indoors due to their energy requirement.

**Ovitrap (OVT)**. This trap consists of a black plastic container filled with 300ml of hay infusion. A wooden paddle held vertically on the wall serves as substrate for mosquito oviposition [4]. After a few days paddles are removed and laid eggs counted. In this study, in each 1 km^2^ area, 120 ovitraps were installed and exposed for five days in a place located in a shaded peridomestic environment. The number of ovitraps was based on [14].

**MosquiTRAP (MQT)**. This trap is designed to collect gravid *Ae. aegypti* females [9]. It is made of a matte-black container filled with 300 ml of water, and requires a synthetic attractant (AtrAedes), and an adhesive card. Attracted ovipositing females stick to the adhesive card.

Monthly, 32 MosquiTRAP units/km^2^ were installed, and removed after seven days. The sample size was twice the size recommended by the manufacturer. This choice was based on results from [13] and sought to improve its sensitivity. Identification and counting was carried out in the field, with the help of a magnifying glass, as recommended by the manufacturer. However, identification of these same samples in the laboratory revealed significant differences both in the total numbers of mosquitoes recorded and in the amount of specimens identified as Ae. Aegypti [15]. MosquiTRAPs were introduced in the study only in the second year.

**Larval surveys**. This survey was carried out concomitantly with the Adultrap installation, always in the same dwellings. Briefly, after inspection of all potential breeding sites, samples of mosquito immatures were collected, and brought to the laboratory to be identified up to the species level. The House Index (HI) waa calculated for each 1 km^2^ area.

### Entomological indices

For each trap, the positivity index was defined as the proportion of traps with at least one capture (one mosquito or one egg), relative to the total units installed and successfully retrieved. In the same way, density indexes were calculated dividing the total number of mosquitoes (or eggs) captured in a given area by the total number of inspected traps.

### Meteorological data

Daily meteorological data were obtained from the Brazilian Environmental Information System (SISAM http://sisam.cptec.inpe.br/sisam/). Weekly temperature and precipitation statistics were calculated as mean, minimum and maximum values. For each of these variables, lagged values (l = 0, 1, 2 and 3 weeks), were also calculated, considering the 7 days before trap collection as lag 0.

### Data analysis

**Comparison between positivity and density indices**. Within each city, scatter plots of density versus positivity indices evidenced a nonlinear relationship. This pattern was expected, as positivity indices are bounded to [0, 100] while density indices are unbounded. In other words, as population increases, the probability of ovipositing in an empty trap decreases. To formalize this observation and compare areas, a linear model of the form *Positivity* = *b* * *Density* and a nonlinear asymptotic model of the form Positivity = a*Density/(K+density) were fitted to each trap data from each locality. A likelihood ratio test was used to check if the nonlinear model provided a significantly better fit than the linear model. The likelihood ratio test compares the difference of the likelihoods of the two models (multiplied by two) to a chi-square distribution. As the fitted models for the same trap tended to be similar among cities, further modeling was carried out combining data from all localities into a single model. Model fitting used the nls function in R 2.12.1 [16] and the likelihood ratio test used the function lrtest in library lmtest [17]

**Regression models**. To infer associations between meteorological variables and trap indices, linear regression model with the identity link function was used. The outcome variable was either the density or the positivity index. Separate models were fit to each trap and each city. To attend the assumption of normality, density and positivity indices were square-root transformed before modeling.

Modeling was carried out step-wisely. First, the best lag for each meteorological variable (Tmin_l, Tmed_l, Tmax_l, Rain_l, l = 0, 1, 2 or 3 weeks) was chosen based on the Akaike Information Criterion (AIC). Secondly, the variable Neighborhood (*Neig*, meaning each of the 1km^2^ areas) was included in all models. The variable *Year* (referring to the study year, first or second year) was also included in the studies that lasted two years. This variable accounted for differences in surveillance efficiency between years, an approach similar to [16]. Only in the Southeast cities, NIG and DQC, models did not include Year, as their surveillance lasted one year only. Since residual analysis of the final models suggested significant autocorrelation of residuals at lag 1, a first order autocorrelation term was also added to all models.

Following this procedure, a model for each city was developed containing *Neig*, *Year* and the set of meteorological variables that were significant for at least one trap within that city. With this procedure, for each city, a common model was available for all concomitantly used traps, allowing comparison between trap systems. Interactions between *Neig* and climate and between *Year* and climate were also assessed and found to be significant in some instances. In these cases, the interaction was included in all trap models for that city. With this approach, we were able to check if a given meteorological variable similarly affected all traps within a city, even if non-significantly.

All statistical analysis were carried out using the software R 2.12.1 [17], library mgcv [18]. Data is available in S1 Dataset in Supplementary material.

## Results

### General results

**Figure 3:**
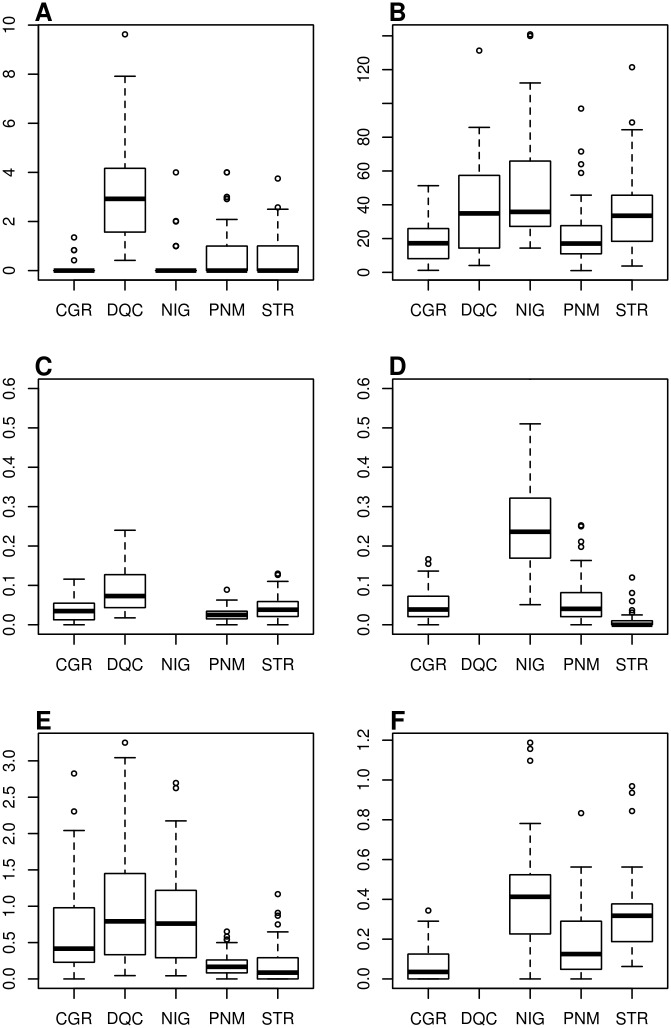
Distribution of house indices (a) and trap density indices (b–f) measured in the five study sites. City legends as in Fig. 2. DQC and NIG were evaluated, respectively, only in the first and in the second year of fieldwork. “Adultrap (1 day)” stands for the original protocol, recommended by the manufacturer, consisting of exposure during 24 hours and employed during the first year; “(Adultrap 4 days)” was the approach used during the second year aiming to improve trap sensitivity.

Fig. 3 shows the range of House Index (HI) and trap density indices measured during the survey. Equivalent panels with trap positivity indices are found in S1 Fig. in S1 Text. Overall, the Southeast cities, NIG and DQC, presented the highest infestation levels among the evaluated sites. Although these two cities have been surveyed in different years, both presented very similar infestation magnitudes according to OVT and BGS measurements—the two traps employed in both cities with the same protocol (see Methods section: in the second year MQT was introduced and ADT protocol was changed). The larval survey detected high premise indices in DQC but failed to detect infestation in NIG. In contrast, according to ADT, infestation was higher in NIG. However, this may have resulted from the increased ADT exposure applied in the second year in NIG (4 days), compared to the 1-day exposure of this trap in the first year, in DQC.

The House Index was consistently low in all cities, except in DQC. The median was always 0, that is, in 50% of months, not a single household was found positive. CGR, which experienced a large dengue outbreak during the survey, exhibited the lowest HI. The traps, on the other hand, rarely produced null indices, pointing to their greater sensitivity in detecting the presence of *Ae. aegypti* in comparison to the larval survey. STR, CGR and PNM tended to present lower infestation indices when compared to DQC and NIG, according to all traps. Ovitraps in CGR and PNM captured the least number of eggs; BGS captured the least number of *Ae. aegypti* in PNM and STR; ADT exposed during 4 days tended to capture more mosquitoes than after only 1 day exposure.

### Relation between density and positivity indices

**Figure 4:**
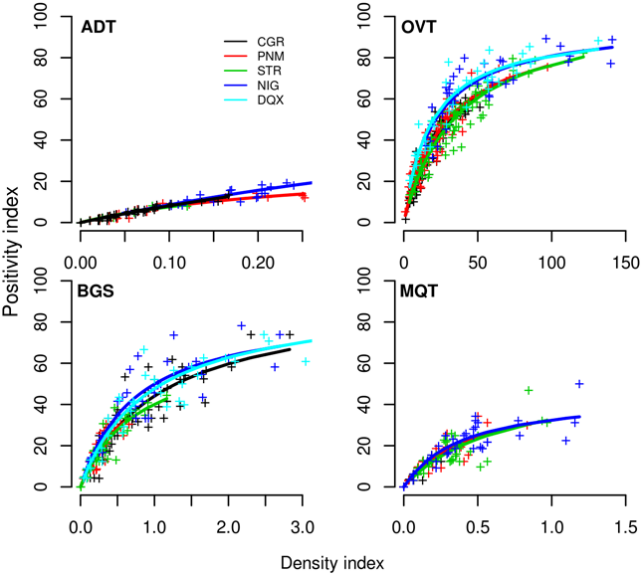
Scatterplots of positivity versus density indices measured by Adultraps (ADT), ovitraps (OVT), BG-Sentinels (BGS) and MosquiTRAPs (MQT) in the five study sites: CGR = Campo Grande, PNM = Parnamirim, STR = Santarém, DQC = Duque de Caxias, NIG = Nova Iguaçu. Symbols: (+) data points; (lines) fitted nonlinear models. See parameters in Table S2 in S1 Text.

Fig. 4 shows the scatterplot of trap positivity versus density indices. The nonlinear model was the best model for all traps but the ADT in Campo Grande (p-value = 0.02) (S2 Table in S1 Text). Remarkably, for each specific trap, the nonlinear relationship between positivity and density was very consistent among cities, despite the differences in climate and mosquito abundance. The parameters of the nonlinear model fitted to the combined data from all cities are shown in Table 1. It is evident from both Fig. 4 and Table 1 that for each trap, positivity indices plateau at different mosquito densities. For example, the ovitrap positivity index tends to plateau at 80%, when egg density exceeds ca. 50 eggs/trap. BGS positivity index shows signs of nonlinear behavior as mosquito density exceeded ca. 1.0 mosquito/trap. MQT positivity never reached values as high as those attained by OVT and BGS. Positive MQT rarely exceeded 40%. Nonlinear relationship between MQT positivity and density indices is stronger when mosquito density is above 0.5 mosquitoes/MQT.

**Table 1:** Point estimates (and their 95% confidence intervals) for the parameters of the linear and the saturation models fitted to trap positivity.

**Trap**	**Nonlinear model**		**Linear model**	**LR**
	**a**	**K**	**b**	**p-value**
**ADT**	97 (79–125)	1.1 (0.8–1.49)	68.7 (66–70)	< 0.001
**OVT**	105 (99–111)	31 (28–35)	1.16 (1.11–1.22)	< 0.001
**BGS**	95 (88–103)	1.08 (0.93–1.24)	36.6 (34.9–38.2)	< 0.001
**MQT**	49 (41–60)	0.5 (0.36–0.72)	44.7 (41.9–47.6)	< 0.001

The results of models fitted by city are found in the Supplement. LR = likelihood ratio test between linear and nonlinear models.

ADT showed less evidence of nonlinear association between positivity and density indices. It was also the trap with the lowest positivity indices, never exceeding 20%. This weak nonlinearity is explained by the fact that each ADT rarely captures more than 1 mosquito, thus, density and positivity indices tend to equate.

### *Ae. aegypti* temporal dynamics

**Figure 5:**
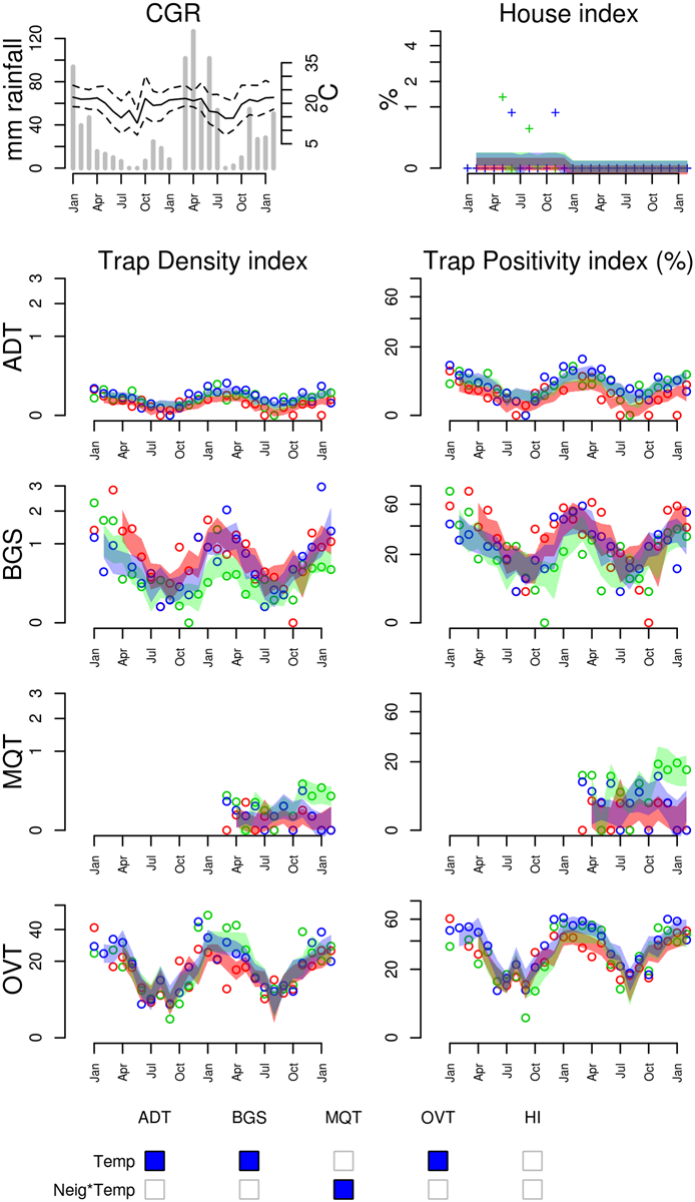
Entomological and meteorological data collected in Campo Grande (CGR) from January 2010 to January 2012. **(Top left)** Meteorological data: (lines) monthly minimum, mean and maximum temperatures, (bars) mean weekly precipitation during each month. **(Top right)** House Index time series of each of the three non-adjacent 1km^2^ neighborhoods (different colors). Colored stripes indicate the 95% predicted range for each area, based on the climate regression model shown in Table S3 in S1 Text. **(From the second row down, left column)** Trap density indices (mean # mosquitoes or eggs / trap) measured by Adultraps (ADT), BG-Sentinel (BGS), MosquiTRAPs (MQT) and ovitraps (OVT). **(From the second row down, right column)** Trap positivity indices (percent of traps with mosquitoes or eggs) measured by ADT, BGS, MQT and OVT. **(Bottom)** Representation of the main patterns observed in the association between trap density indices and meteorological variables: (blue square) positive association; (red square) negative association (p-value < 0.2). Refer to Table S3 in S1 Text for the climate regression model used to define the 95% predicted intervals.

**Figure 6:**
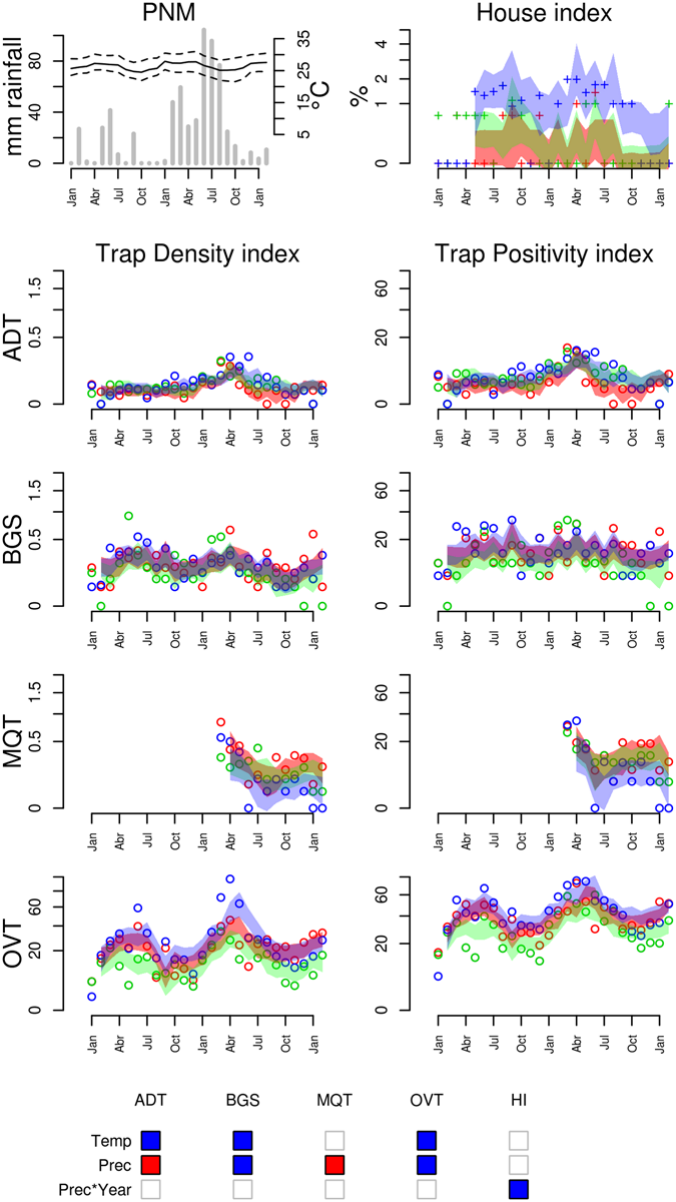
Entomological and meteorological data collected in Parnamirim (PNM), from January 2010 to January 2012. Refer to Fig. 5 for explanation and Table S4 in S1 Text for the climate regression model used to define the 95% predicted intervals.

**Figure 7:**
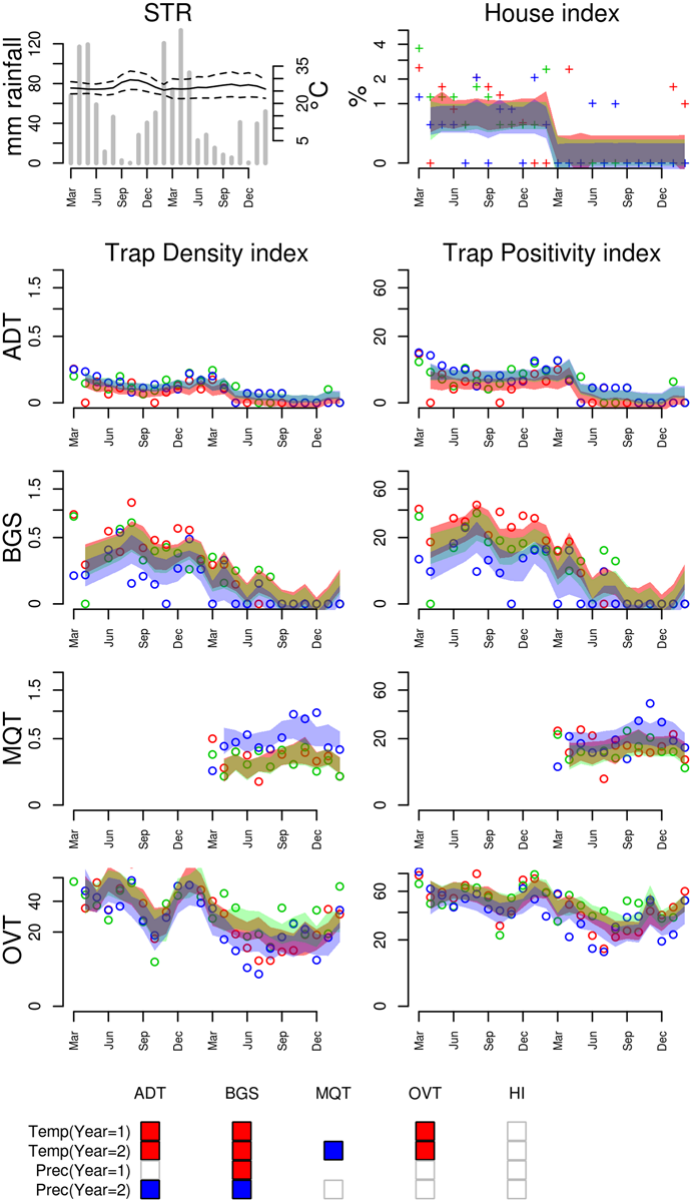
Entomological and meteorological data collected in Santarém (STR), from March 2010 to February 2012. Refer to Fig. 5 for explanation and Table S5 in S1 Text for the climate regression model used to define the 95% predicted intervals.

**Figure 8:**
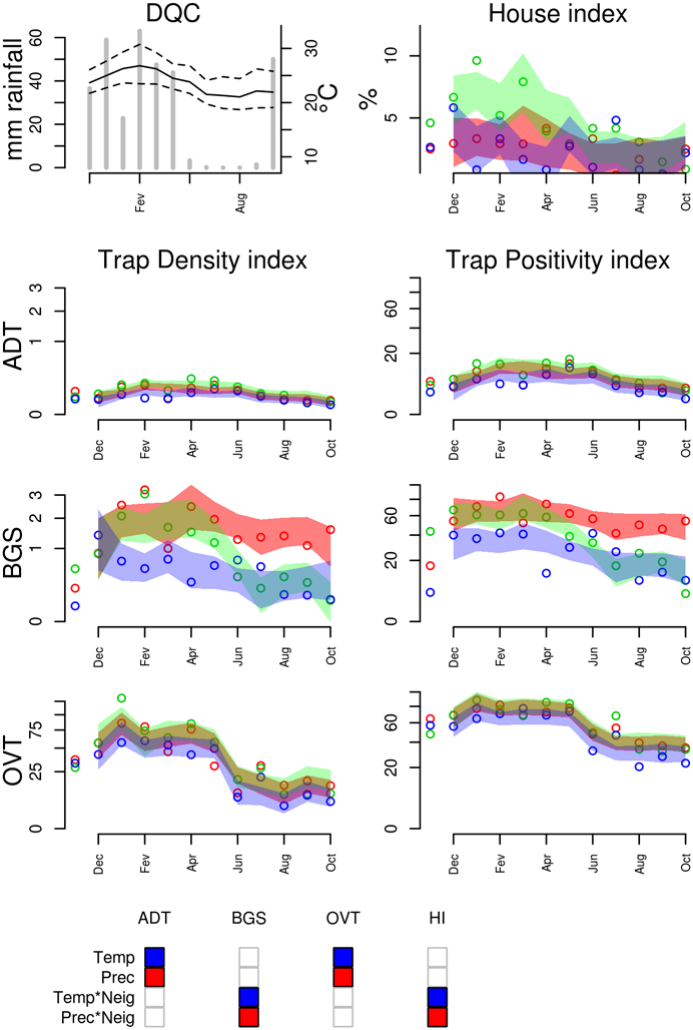
Entomological and meteorological data collected in Duque de Caxias (DQC), from November 2009 to October 2010. Refer to Fig. 5 for explanation and Table S6 in S1 Text for the climate regression model used to define the 95% predicted intervals.

**Figure 9:**
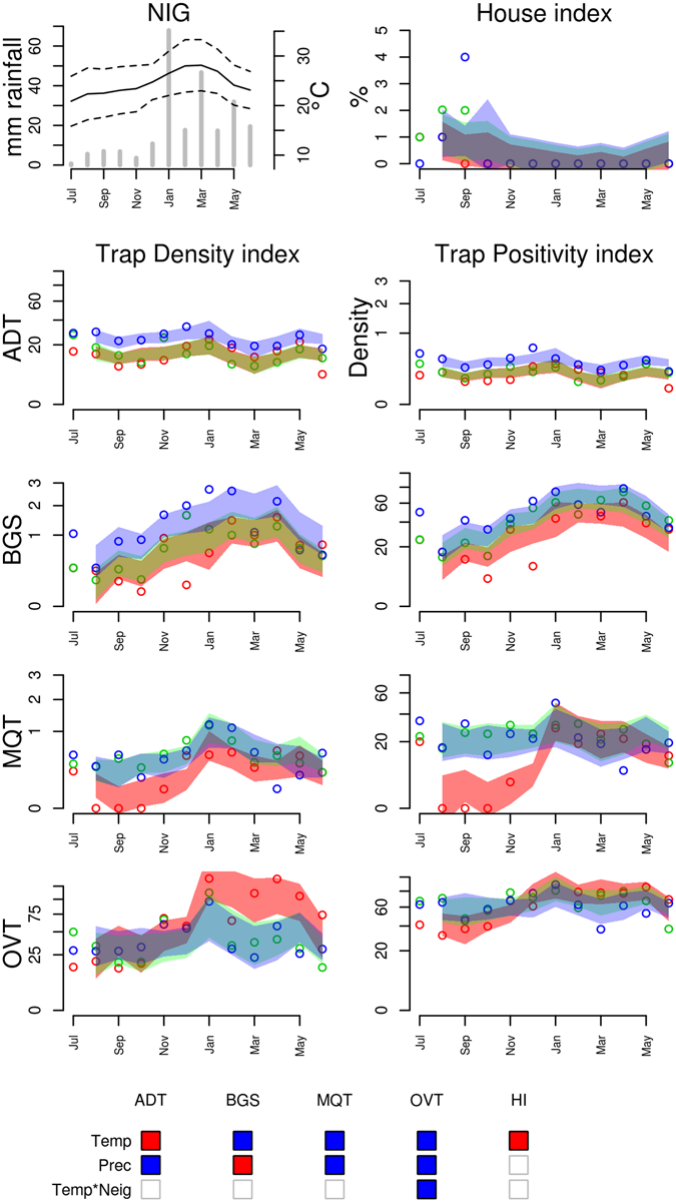
Entomological and meteorological data collected in Nova Iguaçu (NIG), from July 2011 to June 2012. Refer to Fig. 5 for explanation and Table S7 in S1 Text for the climate regression model used to define the 95% predicted intervals.

Figs. 5–9 show the time series of *Ae. aegypti* positivity and density indices using ADT, BGS, MQT and OVT traps for each studied city. House indices and meteorological time series are also shown. Overall, positivity and density indices produced very similar temporal patterns. This agreement is confirmed by the qualitative similarity of the regression models fitted to both indices (S3–S7 Tables in S1 Text). Below, we describe the association between infestation and climate referring only to the density indices, but the conclusions are directly applicable to the positivity indices as well.

***Ae. aegypti* dynamics in Central Brazil (CGR)**. The highland tropical city of Campo Grande has the most marked seasonality among the study sites. The surveyed period consisted of two typical warm-wet summers and two dry-cool winters (Fig. 5). Temperature and precipitation are weakly positively correlated (Pearson’s r = 0.2, p = 0.09). Mosquito density, as measured by ADT, BGS and OVT (used during the 2 years) presented very consistent patterns among study areas and traps, with mosquito population peaking during the summer months, and drastically dropping during the dry winters. *Ae. aegypti* density, as measured by these three traps, is strongly and positively correlated with minimum or mean air temperature at lags 1 week (OVT) or 2 weeks (ADT and BGS) (S3 Table in S1 Text). MQT, included in the study during the second year, presented a similar pattern, but not in all areas. Precipitation also showed correlation with mosquito index, but lost significance in a model with temperature. Among the adult traps, BGS was the most efficient trap, generating indices up to 3 mosquitoes/trap, while ADT and MQT never exceeded 1 mosquito/trap.

The House Index in CGR presented very low values throughout the study, with no seasonal signal. HI was null during the 2010 dengue epidemic, which attacked 3.8% of the population, with a peak incidence of 1105 cases: 10.000 inhabitants in February. Considering an alert threshold of 1 mosquito/trap [19], BGS would launch an alert for this epidemic.

***Ae. aegypti* dynamics in Northeastern Brazil (PNM)**. Differently from CGR, climate in Parnamirim is warm year around, with a temperature regimen that is always favorable to the development of *Ae. aegypti* (Fig. 6). Precipitation usually spreads through the first six months of the year, increasing in intensity during the winter months.

In the course of the study, PNM presented contrasting precipitation regimens, the first year being very dry, with 6 months without precipitation, while the second year was very wet. The house index showed strong spatial heterogeneity, with one site presenting HI > 1% in most of the study, and 2 sites with HI < 1%. No trap confirmed this difference among neighborhoods. This result may indicate variation in the types of breeding sites affecting the immatures’ search success in different areas.

No adult trap ever exceeded 1 mosquito/trap in PNM, as was observed in CGR, suggesting lower infestation. Ovitrap indices, on the other hand, reached higher values in PNM than in CGR.

ADT and OVT detected higher mosquito abundance in the second year than in the first year, but only for ADT this difference between the two years was significant (S4 Table in S1 Text). This is certainly due to the protocol modification implemented for ADT in the second year (although the same is not observed in the other cities). Regression models show that minimum or maximum temperatures at lags 1 or 3 weeks are strong positive predictors of mosquito abundance in Parnamirim, according to BGS, ADT, and OVT. On the other hand, precipitation did not show a consistent association with mosquito density. While OVT and BGS detected positive effects at lag 1 week, ADT and MQT detected weak negative effects at lag 0. ADT, OVT and MQT are traps that attract female mosquitoes searching a place for oviposition. The ADT and MQT’s negative association with precipitation at lag 0 may be explained by the fact that strong precipitation affects flight behavior, does reducing the searching effort. Association with precipitation at lag 1, detected by the BGS, may reflect direct effects of precipitation on egg hatching and subsequent adult recruitment.

***Ae. aegypti* dynamics in Northern Brazil (STR)**. Santarém contrasts with the previous cities by its constant high temperatures and its monsoon season (Fig. 7). The two years of study were very similar in climate but very different in mosquito abundance. The second year presented significantly less mosquitoes than the first year, and this was attributed to the implementation of a new vector control program during the second year. This difference between years, perceived by all methods, was treated in the regression model by fitting the climate effects within each year (S5 Table in S1 Text).

Although small, a temperature variation does occur in Santarém, in response to clouding. In months with high precipitation, less solar incidence reduces the temperature a few degrees. This effect causes a negative correlation between precipitation and temperature (Pearson’s r = -0.19, p = 0.1). The regression models identified a negative effect of minimum temperature at lag 0 on mosquito abundance in Santarém, according to ADT, BGS and OVT. Positive association with precipitation was found in the second year (ADT and BGS), while a negative association was found in the first year (BGS). The only trap giving a very different seasonal signal was MQT, which detected greater mosquito abundance during the dry season instead of the wet season. A study conducted in Manaus (similar climate) comparing BGS and MQT found the same results: while BGS tended to find more mosquitoes during the wet season, MQT found more in the dry season [20]. One possible explanation for MQT increased capture during the dry season could be the effect of reducing the number of competing breeding sites; however, as the same effect was not observed in the OVT and ADT time series, this effect might be related to other causes, for example, the efficiency of the adhesive card or other technical features of the MQT.

***Ae. aegypti* dynamics in Southeastern Brazil (DQC and NIG)**. Duque de Caxias (DQC), where field work was conducted in the first year, suffered an extreme flooding event during the summer which caused severe disruption of the city’s services (Fig. 8). In DQC, all traps (ADT, OVT and BGS) as well as the HI, detected higher mosquito abundance during the summer months with a positive association with minimum temperature at lags 2 or 3 (S6 Table in S1 Text). Precipitation at lag 0 was negatively associated with infestation, likely due to the flooding event. This seasonal pattern is consistent throughout the whole study area as measured by ADT and OVT, but appears only in some neighborhoods, according to BGS. MQT was not used in Duque de Caxias.

Nova Iguaçu (NIG), the adjacent city, entered the study in the second year, replacing DQC. Climate is similar in both cities, but during the second year, no flooding events were recorded (Fig. 9). Trap indices in NIG tended to increase during the summer months. BGS, MQT and OVT detected strong association with temperature at lags 1 to 3 (S7 Table in S1 Text). On the other hand, ADT and HI behaved differently, with a negative association between trap positivity and temperature. These two measurements were taken exactly in the same dwellings. An interaction between temperature and area was detected by the ovitrap and the MosquiTRAP, but the latter was not significant. Association between precipitation and mosquito abundance was inconsistent, while BGS detected a significant negative association, all the other traps detected positive associations.

## Discussion

Dengue vector surveillance is a time and resource consuming activity in many tropical countries. In Brazil, it is estimated that more than 300 million dollars are spent in this activity every year. In this country and many other dengue endemic countries, surveillance protocols are based on larval inspections [21]. Larval surveys are good for identifying key containers, but often fail in providing fast and localized measurements of mosquito abundance. Traps are presented as a complementary approach for dengue vector surveillance and several studies have tested their sensitivity and efficacy. This study expands this discussion by presenting results from a large scale project including five dengue endemic cities simultaneously monitored by four different trap schemes besides the standard immature mosquito survey. Our goal was to reproduce, as well as possible, the real conditions to be faced by a surveillance program using the human and infrastructure resources present in each city. It is important to note, however, that we chose Brazilian municipalities with a prominent record of dengue vector control initiatives and a better than average infrastructure.

Overall, during the study, the house indices rarely reached values above the 4% alert threshold, the only exception being DQC. Still, all cities recorded dengue transmission during the study period and CGR reported a large epidemic. For many reasons, the standard larval survey was not capable to issue proper alerts. For example, failure to detect larval breeding sites despite the presence of high trap indices may indicate that female mosquitoes are choosing more cryptic places to deposit their eggs, such as clogged rain gutters and pipes [7].

In comparison, all traps detected increased mosquito infestation during the dengue transmission seasons, indicating their ability to detect mosquito density variation. Previous studies have compared larval surveys to ovitraps finding the latter more sensitive [7, 22–24], and cost-effective at low mosquito densities [25]. This greater sensitivity of traps to mosquito density variation is probably due to their ability to cover more than one premise while immature surveys only encompass those houses included in the sample. In CGR, BGS was the only adult trap to capture more than 1 mosquito/trap during the dengue epidemic. The other traps exhibited less efficient mosquito collection, but note that MQT was not in use during this period.

This is not the first study to compare traps. Ovitraps were more sensitive than MosquiTRAPs in the low infestation season in Belo Horizonte, Brazil [26] and Pedro Leopoldo, Brazil [19]. In mark-release-recapture experiments, MosquiTRAPs captured more marked mosquitoes than Adultraps [27]. In Rio de Janeiro, Brazil, an experiment with the concomitant distribution of ovitraps and MosquiTRAPs, resulted in greater ovitrap positivity indices [14, 28]. Despite these variations, in general traps perform better than the larval surveys.

### Positivity versus density indices

One practical question that arises in a trap based surveillance program is the possibility of using positivity as a proxy for the more time consuming density indices. If both measures were linearly correlated, this approximation would be easily defended. However, our results suggest that under field conditions, this does not occur for any of the traps. This is attributed to the aggregated spatial distribution of *Ae. aegypti*, typical of many insects [29, 30]. Mogi et al [25] and Ho et al [24] found that ovitrap data fit reasonably well to the empirical model developed by Gerrard and Chaing [31] for aggregated mosquito distributions. Differently from these authors, here we used the Michaelis-Menten model to represent this association. One advantage of the Michaelis-Menten model is the interpretation of its parameters. The parameter“a” stands for the maximum positivity, that is, the positivity index as density tends to infinite; the parameter“K” stands for the mosquito density when positivity index is“a“/2. In other words, above positivity = a/2, the association between positivity and density tend to vanish as positivity saturates.

*Ae. aegypti* positivity-density relationship varied among traps but was consistent among cities, despite the differences in climate (Fig. 4). These intrinsic sensitivity differences among traps have surveillance implications, that is, premises within the study area can be considered negative or positive depending on the method. Reasons for this difference can be attributed to trap attractiveness (more attractive traps covering larger geographical areas), and to different target populations.

A trap based surveillance program should be able to launch alerts if the trap index crosses a predefined threshold. Ideally, such threshold should be estimated based on the minimum mosquito density required to sustain dengue transmission. However, this absolute number is not known for any of the studied traps. A practical alternative would be to define an empirical value, based on the range of densities observed in the past during dengue transmission seasons. For instance, in Australia, control measures are strengthened when sticky ovitrap density index are above 1 mosquito/trap [32]; in Brazil, the MI-Dengue surveillance service uses a threshold of 2 mosquitoes/MQT to launch an alert signal [33].

Now, let’s suppose this threshold is a certain quantity of mosquitoes/trap (m_c_). A trap positivity index will be a good proxy for this density index only if m_c_ < K. Under this condition, the positivity-density conversion is done in a situation where positivity and density indices show high covariation. If m_c_ > K, on the other hand, decision is done in a parameter region where positivity index has low precision and small variations in positivity may represent large variations in density. Applying this rule to the traps under study, we conclude that for ADT, the condition m_c_ < K is likely to be met in most conditions, that is, ADT positivity index is a good proxy for ADT density index. This is a straightforward conclusion anyway, since ADT often captures no more than one mosquito per trap. In the other extreme, OVT’s estimated K was 28–36 eggs/trap which is relatively low compared to the observed range of density observed. Thus, the ovitrap density index will only be reasonably approximated by the positivity index if *mc* is equal to low to moderate mosquito densities. The same seems to apply to BGS and MQT (see S2 Table in S1 Text). In the absence of a well established threshold, density measurements are preferable, for more sensitive traps, as BGT and OVT.

### Different climates

*Aedes aegypti* geographical range is roughly limited to the intertropical area (35°N–35°S), where temperature is mostly above 10°C year around. Our study covered several latitudes within this range and showed that different tropical climates have distinct seasonal patterns of mosquito abundance. *Aedes aegypti* population oscillations were clearly related to seasonality in the tropical climates, even at the equator. Several studies have analyzed the effect of temperature, precipitation and even relative humidity on mosquito abundance in different parts of the globe, with sometimes inconsistent results [7, 14, 22, 24, 26, 29, 32, 34–41]. Compared to other tropical climates, the Af type has the weaker association between mosquito infestation and temperature, although association with precipitation remains significant.

Precipitation sometimes exerts negative effects on mosquito abundance. We found situations in which heavy precipitation events may have disturbed the traps and altered mosquito flight behavior (DQC, PNM and STR). This negative association has also been observed in Selangor, Malaysia [34]. As precipitation contributes to the creation of new breeding sites, it may also reduce the attractiveness of oviposition traps, causing low catching rates. In contrast, positive association of precipitation and mosquito abundance was found in CGR and NIG. Both are cities with typical tropical wet summers and dry winters. It is also relevant that the high correlation between temperature and precipitation makes it difficult to isolate the effects of each factor by regression analysis. The negative association between temperature and mosquito abundance in the equatorial STR was not expected. Since in this city lower temperature is associated with higher precipitation, this result was interpreted as an indirect measurement of precipitation.

### Traps as surveillance tools

Sensitivity to mosquito density variation is an important feature of a trap based surveillance scheme. Among the studied cities, CGR represents the best scenario to test such sensitivity, as it is the only one with strong seasonal variation. We found that Ovitraps, Adultraps and BG Sentinels were all capable of detecting large mosquito variations throughout the year, in a consistent way. This feature is less evident for MosquiTRAPs, which showed strong variation between the three sites. One disclaimer, though, is that MosquiTRAPs were used for a single year and further studies should be done to confirm these initial results.

Many authors have supported the use of adult traps in the place of ovitraps for surveillance. Our results show that ovitraps, although not measuring directly the adult population, do capture its variation very well. Actually, of all traps, ovitrap was the one with best sensitivity (never presented null indices), and strongest association with climate, and consistently followed the adult mosquito patterns detected by the adult traps. These results confirm the usefulness of ovitraps for *Ae. aegypti* surveillance, even if it does not produce direct indices of adult mosquito abundance.

At last, independently of the trap chosen, it should be considered that, although to a lesser extent than larval surveys, trap based indices are still dependent on the work quality of the field personnel. This is especially relevant in the scope of a broad and regular surveillance program and relates to aspects such as traps installation and collection, as well as specimens counting or identification.

## Conclusion

This study was carried out to support the development of trap based surveillance programs in dengue endemic countries. Our main conclusions are that all investigated traps are valuable tools and could be considered in combination with vector control strategies to improve our response to dengue and other diseases transmitted by *Ae. aegypti*. Household larval surveys and trap based surveillance systems are not interchangeable approaches though. Household surveys are required for the identification of the major mosquito breeding sites in a given locality. This allows the design of adequate control or elimination strategies. Traps are useful for monitoring adult infestation levels and the impact of control strategies. Used together, they synergistically optimize both surveillance, prevention and control. Future studies should assess the cost-benefit of such integrated strategies. Other features should be also evaluated before choosing a trap for surveillance: specificity, low cost, ease of distribution, a consistent sampling profile [3]. This will be subject of future studies.

## Supporting Information

S1 Text Contains detailed tables and figures as following. **S1 Fig.** Boxplot showing the distribution of house indices (a) and trap positivity indices (b–f) measured in the five study sites City legends as in Fig. 2 DQC and NIG were evaluated, respectively, only in the first and in the second year of fieldwork. “Adultrap (1 day)” stands for the original protocol, recommended by the manufacturer, consisting of exposure during 24 hours and employed during the first year; “(Adultrap 4 days)” was the approach used during the second year aiming to improve trap sensitivity **S1–S7 Tables**. Description of the study area and fitted linear and nonlinear models.
(ODT)

S1 Dataset Zip file containing the dataset used in this study. It consists of four.csv files, one for each trap ADT.csv contains the Adultrap data, BGS.csv contains the BG Sentinel data, MQT.csv contains the Mosquitrap data, OVT.csv contains the Ovitrap data Each file has the following variables: year (1 or 2), city (one of the five cities), Neig (3 neighborhoods per city), Month, HI (House index), di (density index), ADTpi (positivty index), date (date), Prec_0, Prec_1, Prec_2, Prec_3 (mm rainfall at lag 0 to 3 weeks), T_Min_0, T_Min_1, T_Min_2, T_Min_3 (mean minimum temperature at lag 0 to 3 weeks), T_Med_0, T_Med_1, T_Med_2, T_Med_3 (mean daily temperature at lag 0 to 3 weeks), T_Max_0, T_Max_1, T_Max_2, T_Max_3 (mean maximum temperature at lag 0 to 3 weeks).
(ZIP)
