# Supplementary material for: Surveillance of Aedes aegypti: Comparison of House Index with Four Alternative Traps
Source: PLoS Negl Trop Dis. 2015 Feb 10;9(2):e0003475. doi: 10.1371/journal.pntd.0003475 (PMC4323346; doi:10.1371/journal.pntd.0003475)
Supplement: Supplementary file 1 [file pntd.0003475.s001.odt]

Support information material: Codeço et al. Surveillance of Aedes aegypti: comparison of house index with four alternative traps

Table S1. Some descriptors of the five cities included in the entomological survey.

| Region | City | Area (km2) | Population (x 1000) | 12 months survey beginning dates |
| --- | --- | --- | --- | --- |
|  |  |  |  | Year 1 | Year 2 |
| North | Santarém (STR) | 22,886 | 294 | Mar (2010) | Mar (2011) |
| Northeat | Parnamirim (PNM) | 123 | 202 | Dec (2009) | Dec (2010) |
| Southeast | Duque de Caxias (DQC) | 467 | 855 | Nov (2009) | - |
| Southeast | Nova Iguaçu (NIG) | 521 | 796 | - | Jul (2011) |
| Central-West | Campo Grande (CGR) | 8,093 | 787 | Dec (2009) | Dec (2011) |

Figure S1. Distribution of house indices (a) and trap positivity indices (b-f) measured in the five study sites. City legends as in Figure 2. 

I- Positivity X density indices

Table S2. Point estimates and 95 confidence intervals of the parameters of the linear model (Positivity = b * Density) and a nonlinear model (Positivity = a Density /(K + Density)) fitted to positivity X density indices collected by each trap in each city. LR refers to the result of the likelihood test for comparison between linear and nonlinear models. ADT, OVT, BGS and MQT refer to the four traps used: Adultrap, Ovitrap, BG Sentinel and MosquiTRAP.  Cities: CGR = Campo Grande; PNM = Parnamirim; STR = Santarem; NIG = Nova Iguaçu; DQC = Duque de Caxias.  

| City | Trap | Nonlinear model | Linear model | LR |
| --- | --- | --- | --- | --- |
|  |  | a | K | b | p-value |
| CGR | ADT | 73 (41-801) | 0.8 (0.4-9.8) | 67 (61-73) | 0.024 |
|  | OVT | 106 (90-128) | 34 (26-47) | 1.63 (1.54-1.72) | < 0.001 |
|  | BGS | 92 (78-112) | 1.1 (0.8-1.56) | 37.2 (34-40) | < 0.001 |
|  | MQT | 42 (28-83) | 0.41 (0.22-1) | 67.3 (61-73) | < 0.001 |
|  |  |  |  |  |  |
| PNM | ADT | 29 (23-39) | 0.27 (0.19-0.4) | 51 (45-58) | < 0.001 |
|  | OVT | 98 (86-113) | 28 (22-37) | 1.44 (1.32-1.56) | < 0.001 |
|  | BGS | 71 (51-115) | 0.75 (0.46-1.4) | 63.8 (59.6-68) | < 0.001 |
|  | MQT | 46 (32-79) | 0.45 (0.22-1) | 51.8 (45.4-58.1) | < 0.001 |
|  |  |  |  |  |  |
| STR | ADT | 25 (18-42) | 0.23 (0.15-0.44) | 44 (38-50) | < 0.001 |
|  | OVT | 104 (92-119) | 36 (27-47) | 1.15 (1.07-1.24) | < 0.001 |
|  | BGS | 72 (57-96) | 0.8 (0.54-1.25) | 49.5 (46-53) | < 0.001 |
|  | MQT | 55 (33-123) | 0.68 (0.26-2.1) | 44.5 (38.9-50.2) | < 0.001 |
|  |  |  |  |  |  |
| NIG | ADT | 25 (18-42) | 0.23 (0.15-0.44) | 42 (36-47) | < 0.001 |
|  | OVT | 97 (88-108) | 20 (14-27) | 0.96 (0.8-1.11) | < 0.001 |
|  | BGS | 87 (72-108) | 0.75 (0.46-1.2) | 37.3 (32.4-42.2) | < 0.001 |
|  | MQT | 45 (34-66) | 0.40 (0.19-0.8) | 41.8 (36.3-47.3) | < 0.001 |
|  |  |  |  |  |  |
| DQC (*) | OVT | 96 (87-105) | 18 (13-24) | 1.14 (0.98-1.29) | <0.001 |
|  | BGS | 92 (77-112) | 0.92 (0.6-1.4) | 32.2 (28-36) | < 0.001 |

(*) DQC was evaluated only on the first year of field work. For this reason data related to MQT, which was introduced to the study on the second year, are missing for this locality. ADT also miss for DQC since results shown derive from 4 days of field-exposure, a protocol employed only on the second year.

II-Regression models for each trap/city

Table S3. Regression models for the entomological indices collected in Campo Grande city (CGR). ADT, OVT, BGS and MQT refer to the four traps used: Adultrap, ovitrap, BG Sentinel and MosquiTRAP, respectively.Temperature was the only significant meteorological variable in the models. In blue, the positive effects with p-value < 0.20 (t-test). R2 = Coefficient of determination. 

|  | Campo Grande: Positivity indices |  |
| --- | --- | --- |
| Variables | lag | HI | lag | ADT | lag | BGS | lag | MQT | lag | OVT |  |
| R2 |  | 0.05 |  | 0.45 |  | 0.36 |  | 0.52 |  | 0.62 |  |
| sq(AR1) |  | -0.15±0.12 |  | 0.21±0.10 |  | 0.32±0.10** |  | -0.28±0.14+ |  | 0.37±0.09** |  |
| Area     A |  | 0.18±0.09 |  | -0.66±0.39+ |  | 0.57±0.90 |  | 2.57±2.2 |  | -0.01±0.67 |  |
| B |  | 0.27±0.09 |  | -0.17±0.39 |  | -0.07±0.88 |  | -6.46±2.24** |  | -0.99±0.67 |  |
| C |  | 0.27±0.09 |  | -0.11±0.39 |  | 0.40±0.89 |  | -1.99±2.44 |  | -0.66±0.68 |  |
| Year |  | -0.12±0.05 |  | 0.20±0.15 |  | 0.44±0.36 |  | - |  | 0.33±0.25 |  |
| Temp |  | 0.001±0.006 | Min, l=2 | 0.09±0.02** | Min, l=2 | 0.16 ±0.05** | Mea, l=2 | -0.06±0.11 | Mea, l=1 | 0.25±0.05** |  |
| Tempx B |  |  |  |  |  |  | Mea, l=2 | 0.58±0.16** |  |  |  |
| Tempx C |  |  |  |  |  |  | Mea, l=2 | 0.27±0.17 |  |  |  |
|  | Density indices |  |
| Variables |  |  | lag | ADT | lag | BGS | lag | MQT | lag | OVT |
| R2 |  |  |  | 0.45 |  | 0.47 |  | 0.48 |  | 0.55 |
| sq(AR1) |  |  |  | 0.15±0.11 |  | 0.38±0.1** |  | -0.3±0.14* |  | 0.42±0.09** |
| Area     A |  |  |  | -0.09±0.04* |  | -0.05±0.16 |  | 0.21±0.27 |  | -1.32±0.66+ |
| B |  |  |  | -0.04±0.04 |  | -0.20±0.15 |  | -0.88±0.2** |  | -1.17±0.66+ |
| C |  |  |  | -0.02±0.04 |  | -0.10±0.15 |  | -0.33±0.30 |  | -1.2±0.66+ |
| Year |  |  |  | 0.03±0.02 |  | 0.08±0.06 |  |  |  | 0.08±0.24 |
| Temp |  |  | Min, l=2 | 0.011 ±0.002** | Min,l=2 | 0.03±0.008** | Mea,l=2 | -0.002±0.01 | Mea, l=1 | 0.26±0.05** |
| Temp x B |  |  |  |  |  |  | Mea,l=2 | 0.07±0.02** |  |  |
| Temp x C |  |  |  |  |  |  | Mea,l=2 | 0.03±0.02 |  |  |

P-values: (+) 0.05 < p < 0.01 ;(*)  0.001 < p < 0.05; (**) p < 0.001. Min, l=2 means Minimum temperature, lag = 2 weeks. Mea=Mean. AR1 = response variable with time lag 1;

Areas A,B,C = each of the 1 km2 study sites.

Table S4. Regression models for Parnamirim city (PNM) including the variables temperature (Temp) and precipitation (Prec). In blue (positive) and red (negative), the estimates

with p-value < 0.20 (t-test).Refer to Table S3 for the acronyms

|  | Parnamirim: Positivity indices |
| --- | --- |
| Variables | lag | HI | lag | ADT | lag | BGS | lag | MQT | lag | OVT |
| R2 |  | 0.40 |  | 0.32 |  | 0.15 |  | 0.30 |  | 0.53 |
| sq(AR1) |  | 0.28±0.12* |  | 0.49±0.10** |  | 0.14±0.11 |  | 0.40±0.15* |  | 0.33±0.08** |
| Area     A |  | 0.63±0.27* |  | -3.44±1.77 |  | 0.31±2.61 |  | 7.87±3.94+ |  | -7.8±3.41* |
| B |  | 0.75±0.28** |  | -3.26±1.77 |  | -0.24±2.61 |  | 7.68±3.95+ |  | -8.3±3.41* |
| C |  | 1.21±0.29*** |  | -3.25±1.77 |  | 0.47±2.61 |  | 7.05±3.96+ |  | -7.46±3.42* |
| Year |  | -0.38±0.15 |  | 0.32±0.18 |  | -0.37±0.29 |  | - |  | 0.04±0.23 |
| Temp | Min, l=0 | 0.02±0.05 | Min, l=3 | 0.15±0.07* | Min, l=1 | 0.12±0.10 | Min, l=0 | -0.23±0.17 | Max, l=1 | 0.40±0.12** |
| Prec | l=1 | -0.008±0.01 | l=0 | -0.003±0.002 | l=1 | 0.018±0.006** | l=0 | -0.013±0.005* | l=0 | 0.006±0.003+ |
| Prec*Year |  | 0.008±0.005 |  |  |  |  |  |  |  |  |
|  | Parnamirim: Density indices |
| Variables |  |  | lag | ADT | lag | BGS | lag | MQT | lag | OVT |
| R2 |  |  |  | 0.33 |  | 0.19 |  | 0.32 |  | 0.52 |
| sq(AR1) |  |  |  | 0.50 ±0.10** |  | 0.21±0.11+ |  | 0.36±0.14* |  | 0.39±0.08** |
| Area     A |  |  |  | -0.40±0.21 |  | -0.40±0.35 |  | -0.94±1.05 |  | -14.1±4.2** |
| B |  |  |  | -0.39±0.21 |  | -0.45±0.35 |  | -0.98±1.05 |  | -14.7±4.28** |
| C |  |  |  | -0.38±0.21 |  | -0.39±0.35 |  | -1.07±1.05 |  | -13.7±4.26** |
| Year |  |  |  | 0.044±0.022 |  | -0.07±0.04+ |  |  |  | -0.24±0.3 |
| Tmin |  |  | Min, l=3 | 0.018±0.009* | Min, l=1 | 0.034±0.015* | Max, l=2 | 0.04±0.03 | Max, l=1 | 0.580.15** |
| Prec |  |  | l=0 | -0.0004±0.0002+ | l=1 | 0.002±0.0008** | l=0 | -0.001±0.0007 | l=1 | 0.007±0.003* |

P-values: (+) 0.05 < p < 0.01 ; (*) 0.001 < p < 0.05; (**) p < 0.001.

Table S5. Regression models for Santarém city (STR), with variables temperature and precipitation by year. In blue (positive) and red (negative), the estimates with p-value < 0.20 (t-test). Refer to Table S3 for the acronyms.

| Santarem | Positivity indices |
| --- | --- |
| Variables | lag | HI | lag | ADT | lag | BGS | lag | MQT | lag | OVT |
| R2 |  | 0.29 |  | 0.58 |  | 0.68 |  | 0.19 |  | 0.45 |
| sq(AR1) |  | -0.06±0.12 |  | -0.04±0.14 |  | 0.13±0.11 |  | 0.06±0.16 |  | 0.23±0.11* |
| Area     A |  | 3.31±2.48 |  | 4.8±3.4 |  | 27±10** |  | -10±5.8+ |  | 11±2.6** |
| B |  | 3.25±2.48 |  | 5.3±3.4 |  | 27±10** |  | -10±5.8+ |  | 11±2.6** |
| C |  | 3.19±2.48 |  | 5.4±3.4 |  | 26±10** |  | -9±5.8 |  | 11±2.6** |
| Temp year=1 | Min, l=2 | -0.096±0.10 | Min, l=2 | -0.14±0.14 | Min, l=0 | -0.93±0.39* |  |  | Max, l=0 | -0.19±0.08* |
| Temp, year=2 | Min, l=2 | -0.14±0.11 | Min, l=2 | -0.22±0.15 | Min, l=0 | -1.22±0.44** | Med, l=2 | 0.50±0.21* | Max, l=0 | -0.23±0.08** |
| Prec year=1 | l=0 | -0.001±0.001 | l=0 | 0.001±0.002 | l=3 | -0.02±0.01** | l=3 |  | l=0 | 0.004±0.003 |
| Prec year=2 | l=0 | 0.001±0.002 | l=0 | 0.016±0.003** | l=3 | 0.02±0.008** | l=3 | 0.005±0.003 | l=0 | 0.005±0.003 |
|  | Density indices |
| Variables |  |  | lag | ADT | lag | BGS | lag | MQT | lag | OVT |
| R2 |  |  |  | 0.60 |  | 0.64 |  | 0.56 |  | 0.51 |
| sq(AR1) |  |  |  | -0.008±1.38 |  | 0.003±0.12 |  | -0.09±0.15 |  | 0.30±0.10** |
| Area     A |  |  |  | 0.99±0.35** |  | 4.6±1.4** |  | -1.14±0.56+ |  | 26.4±5.8** |
| B |  |  |  | 1.05±0.35** |  | 4.6±1.4** |  | -1.15±0.56+ |  | 26.6±5.8** |
| C |  |  |  | 1.05±0.35±** |  | 4.4±1.4** |  | -0.85±0.56 |  | 26.0±5.8** |
| Temp year=1 |  |  | Min, l=0 | -0.034±0.013* | Min, l=0 | -0.16±0.05** |  | - | Min, l=0 | -0.88±0.23** |
| Temp, year=2 |  |  | Min, l=0 | -0.045±0.015** | Min, l=0 | -0.20±0.06** | Max, l=2 | 0.052±0.018** | Min, l=0 | -1.04±0.25** |
| Prec year=1 |  |  | l=0 | -0.000±0.00 | l=3 | -0.004±0.001** |  | - | l=0 | -0.006±0.003 |
| Prec year=2 |  |  | l=0 | 0.002±0.0004** | l=3 | 0.002±0.001* | l=0 | 0.0005±0.0004 | l=0 | -0.001±0.004 |

P-values: (+) 0.05 < p < 0.01 ; (*) 0.001 < p < 0.05; (**) p < 0.001

Table S6. Regression models for Duque de Caxias city (DQC) with variables temperature and precipitation. In blue (positive) and red (negative), the estimates with p-value < 0.20 (t-test). Refer to Table S3 for the acronyms.

| Duque de Caxias | Positivity indices |
| --- | --- |
| Variables | lag | HI | lag | ADT | lag | BGS | lag | MQT | lag | OVT |
| R2 |  | 0.54 |  | 0.57 |  | 0.73 |  |  |  | 0.78 |
| sq(AR1) |  | -0.16±0.17 |  | 0.37±0.14* |  | 0.08±0.18 |  |  |  | 0.08±0.13 |
| Area     A |  | -4.18±5.64 |  | -2.41±0.92* |  | 1.08±3.21 |  |  |  | -5.7±1.4** |
| B |  | -16±5.64** |  | -2.26±0.92 |  | -12.06±3.56** |  |  |  | -5.6±1.4** |
| C |  | -9.57±5.64+ |  | -2.68±0.92* |  | -2.6±3.26 |  |  |  | -6.3±1.4** |
| Temp | Min, l=1 | 0.32±0.27 | Min, l=3 | 0.20±0.04*** | Min, l=2 | 0.28±0.16 |  |  | Min, l=2 | 0.59±0.09** |
| Prec | l=2 | 0.01±0.02 | l=0 | -0.007±0.003+ | l=0 | -0.008±0.013 |  |  | l=1 | -0.008±0.004+ |
| Temp*B |  | 0.68±0.38+ |  |  |  | 0.53±0.22* |  |  |  |  |
| Temp*C |  | 0.28±0.38 |  |  |  | 0.04±0.21 |  |  |  |  |
| Prec*B |  | 0.006±0.032 |  |  |  | 0.006±0.01 |  |  |  |  |
| Prec*C |  | -0.059±0.032+ |  |  |  | 0.018±0.01 |  |  |  |  |
| Duque de Caxias | Density indices |
| Variables |  |  | lag | ADT | lag | BGS | lag | MQT | lag | OVT |
| R2 |  |  |  | 0.59 |  | 0.72 |  |  |  | 0.82 |
| sq(AR1) |  |  |  | 0.38±0.14* |  | -0.09±0.17 |  |  |  | -0.26±0.14+ |
| Area     A |  |  |  | -0.31±0.11** |  | -0.3±0.76 |  |  |  | -21±2.9** |
| B |  |  |  | -0.28±0.11* |  | -3.9±0.92** |  |  |  | -20±2.9** |
| C |  |  |  | -0.34±0.11** |  | -0.52±0.78 |  |  |  | -22±2.9** |
| Temp |  |  | Min, l=3 | 0.023±0.005** | Min, l=2 | 0.08±0.04* |  |  | Min, l=2 | 1.34±0.17** |
| Prec |  |  | l=0 | -0.0009±0.0004* | l=0 | -0.006±0.003* |  |  | l=1 | -0.01±0.006* |
| Temp*B |  |  |  |  |  | 0.14±0.05* |  |  |  |  |
| Temp*C |  |  |  |  |  | -0.028±0.05) |  |  |  |  |
| Prec*B |  |  |  |  |  | 0.0009±0.004 |  |  |  |  |
| Prec*C |  |  |  |  |  | 0.01±0.003* |  |  |  |  |

P-values: (+) 0.05 < p < 0.01 ; (*) 0.001 < p < 0.05; (**)p < 0.001

Table S7. Regression models for Nova Iguaçu city (NIG) with variables temperature and precipitation. In blue (positive) and red (negative), the estimates with p-value < 0.20 (t-test). Refer to Table S3 for the acronyms.

| Nova Iguaçu | Positivity indices |
| --- | --- |
| Variables | lag | HI | lag | ADT | lag | BGS | lag | MQT | lag | OVT |
| R2 |  | 0.14 |  | 0.56 |  | 0.72 |  | 0.58 |  | 0.40 |
| sq(AR1) |  | 0.05±0.19 |  | 0.02±0.16 |  | 0.04±0.13 |  | 0.04±0.16 |  | -0.03±0.2 |
| Area     A |  | 2.93±1.40 |  | 6.87±1.4*** |  | -5.38±1.58 |  | -9.25±3.02** |  | -1.63±2.58 |
| B |  | 3.19±1.42 |  | 6.87±1.4*** |  | -4.36±1.60 |  | 4.96±2.74+ |  | 5.83±2.84+ |
| C |  | 3.28±1.42 |  | 8.05±1.51*** |  | -3.98±1.61 |  | 5.10±2.78+ |  | 8.63±2.96** |
| Temp | Min, l=2 | -0.14±0.07 | Min, l=2 | -0.12±0.04** | Min, l=2 | 0.52±0.08** | Min, l=3 | 0.58±0.16** | Min,l=2 | 0.48±0.17** |
| Prec | l=3 | -0.003±0.006 | l=3 | 0.01±0.004* | l=1 | 0.001±0.007 | l=2 | 0.014±0.007+ | l=2 | 0.012±0.005* |
| Temp*B |  |  |  |  |  |  |  | -0.61±0.19** |  | -0.37±0.19+ |
| Temp*C |  |  |  |  |  |  |  | -0.63±0.20** |  | -0.52±0.20* |
|  | Density indices |
| Variables |  |  | lag | ADT | lag | BGS | lag | MQT | lag | OVT |
| R2 |  |  |  | 0.55 |  | 0.63 |  | 0.56 |  | 0.52 |
| sq(AR1) |  |  |  | -0.03±0.17 |  | 0.06±0.17 |  | 0.34±0.15 |  | 0.003±0.16 |
| Area     A |  |  |  | 0.86±0.18*** |  | -1.56±0.39*** |  | -0.37±0.34 |  | -7.39±4.25+ |
| B |  |  |  | 0.87±0.18*** |  | -1.50±0.39*** |  | -0.2±0.33 |  | 3.06±4.12 |
| C |  |  |  | 1.03±0.20*** |  | -1.21±0.39*** |  | -0.22±0.33 |  | 6.15±4.14 |
| Temp |  |  | Max, l=1 | -0.016±0.005** | Min, l=2 | 0.116±0.02*** | Min,l=0 | 0.02±0.017* | Min, l=3 | 0.74±0.23** |
| Prec |  |  | l=3 | 0.0018±0.0005** | l=1 | -0.002±0.002 | l=2 | 0.003±0.001** | l=2 | 0.026±0.011* |
| Temp*B |  |  |  |  |  |  |  |  |  | -0.61±0.28* |
| Temp*C |  |  |  |  |  |  |  |  |  | -0.75±0.28* |

 P-values: (+)0.05 < p < 0.01 ; (*) 0.001 < p < 0.05; (**) p < 0.001.
